# Preoperative CT markers and poor discharge functional status after burr-hole drainage for chronic subdural hematoma: a retrospective cohort study

**DOI:** 10.3389/fneur.2026.1820077

**Published:** 2026-06-09

**Authors:** Jihong He, Piqiang Qi, Xiangbin Liu, Changfeng Wang, Yijun Zeng

**Affiliations:** Department of Neurosurgery, Chengdu Pidu District People's Hospital/The Third Affiliated Hospital of Chengdu Medical College, Chengdu, China

**Keywords:** burr-hole drainage, chronic subdural hematoma, discharge functional status, hematoma thickness, midline shift, modified Rankin Scale, prediction model

## Abstract

**Background:**

Chronic subdural hematoma (CSDH) is among the most common neurosurgical conditions in older adults, yet preoperative predictors of short-term functional status after surgical evacuation remain incompletely defined. In this exploratory study we evaluated whether preoperative computed tomography (CT) markers—specifically hematoma thickness and midline shift—are associated with poor discharge functional status after burr-hole drainage for CSDH, after adjustment for established clinical factors.

**Methods:**

We retrospectively analyzed 260 consecutive adult patients who underwent burr-hole drainage for CSDH at a single institution between January 2018 and December 2023. Because of the retrospective design and absence of prospective registration, the findings were interpreted as exploratory. The primary outcome was poor discharge functional status, defined as a modified Rankin Scale (mRS) score ≥3 at hospital discharge. Multivariable logistic regression identified factors independently associated with the primary outcome. Restricted cubic spline (RCS) analysis examined dose–response relationships. Model performance was characterized by discrimination (area under the receiver operating characteristic curve [AUC] with bootstrap optimism correction), calibration (Hosmer–Lemeshow test and calibration plot), Brier score, and decision curve analysis (DCA) across nested models.

**Results:**

Among 260 patients (mean age 72.9 ± 9.1 years; 70.4% male), 66 (25.4%) had poor discharge functional status. In the full multivariable model, midline shift (adjusted odds ratio [aOR] 1.11, 95% CI 1.01–1.22; *p* = 0.036), pre-hospital mRS (aOR 1.39, 95% CI 1.05–1.85; *p* = 0.022) and age (aOR 1.08, 95% CI 1.04–1.12; *p* < 0.001) were independently associated with the primary outcome. Hematoma thickness was numerically positively associated with poor outcome but did not meet the predefined two-sided *p* < 0.05 threshold (aOR 1.05, 95% CI 1.00–1.11; *p* = 0.067). RCS analysis was consistent with approximately linear dose–response relationships for hematoma thickness (*P*non-linearity = 0.654) and midline shift (*P*non-linearity = 0.094). The full model achieved an apparent AUC of 0.733, with bootstrap-corrected AUC of 0.721 (optimism = 0.012). Calibration was acceptable (Hosmer–Lemeshow *p* = 0.42; Brier score 0.168), and DCA showed positive net benefit across threshold probabilities of approximately 10–45%.

**Conclusion:**

In this single-center retrospective cohort, midline shift, pre-hospital functional status and age were independently associated with poor discharge functional status after burr-hole drainage for CSDH. These exploratory, hypothesis-generating findings may assist preoperative risk stratification, patient and family counseling, postoperative monitoring intensity, and early rehabilitation planning, but should not be used as a stand-alone basis for treatment decisions. External validation in independent multicenter cohorts using longitudinal outcome scales (e.g., 3- or 6-month mRS or Glasgow Outcome Scale Extended) is required before clinical implementation.

## 1 Introduction

Chronic subdural hematoma (CSDH) is among the most frequently encountered neurosurgical conditions, with an incidence that has been steadily rising in parallel with population aging and the increasing use of anticoagulant and antiplatelet therapies (1, 2). Current estimates suggest an annual incidence of 8–14 per 100,000 in the general population, rising to 58–80 per 100,000 among individuals aged 70 years and older (3, 4). Although CSDH has traditionally been considered a benign and surgically curable condition, accumulating evidence indicates that a substantial proportion of patients experience persistent functional disability after surgical evacuation, with rates of poor discharge functional status ranging from 15 to 35% (5, 6). Burr-hole drainage remains the most widely performed first-line surgical treatment, demonstrating favorable recurrence rates and procedural safety profiles compared with craniotomy (7).

Identifying preoperative predictors of functional status is essential for informed clinical communication, structured patient counseling, calibration of postoperative monitoring intensity, and resource allocation. Several clinical factors have been established as prognostic indicators, including advanced age, pre-existing functional disability (measured by the modified Rankin Scale [mRS]), neurological deficit at presentation, and anticoagulant use (8, 9). However, the role of preoperative radiological burden—encompassing hematoma thickness, midline shift, and overall hematoma volume—as an independent correlate of functional status remains a subject of active investigation. Some studies have reported significant associations between radiological parameters and outcome (10, 11), while others have found these variables to be non-predictive after adjustment for clinical confounders (12). Furthermore, prior investigations have seldom examined whether the relationship between radiological burden and outcome follows a linear or threshold-based pattern, limiting the translation of imaging measurements into clinically actionable risk estimates.

To address these knowledge gaps, we conducted a retrospective cohort study in a consecutive series of patients undergoing burr-hole drainage for CSDH. The primary objective was to evaluate whether preoperative hematoma thickness and midline shift are independently associated with poor discharge functional status (mRS ≥ 3 at discharge), after adjustment for established clinical confounders. Secondary objectives included characterizing the dose–response relationship between radiological burden and outcome using restricted cubic spline analysis, and assessing the incremental predictive value of radiological variables beyond clinical factors using nested model comparisons with comprehensive performance evaluation including internal validation, calibration, and decision curve analysis. Given the absence of a pre-specified statistical analysis plan, the analysis was framed as exploratory from the outset. We hypothesized that greater preoperative radiological burden would be associated with worse discharge functional status in a dose-dependent manner (13).

## 2 Methods

### 2.1 Study design and protocol

This was a single-center retrospective observational cohort study conducted at the Department of Neurosurgery, Pidu District People’s Hospital (The Third Affiliated Hospital of Chengdu Medical College), Chengdu, China. The study protocol was reviewed and approved by the institutional Medical Ethics Committee (Approval No. IRB-25-57; date of approval: May 26, 2025). The Committee waived the requirement for individual written informed consent because the analysis used de-identified data extracted from existing medical records and posed no more than minimal risk to participants.

The study was not prospectively registered in a public clinical study registry, and no pre-established statistical analysis plan was filed before data extraction. Variable definitions, candidate predictors, the modeling strategy and the validation procedures described below were specified by the investigator team before formal regression modeling was performed; nevertheless, in the absence of formal pre-registration we cannot fully exclude analytical flexibility, and all results should be regarded as exploratory and hypothesis-generating. This issue is explicitly acknowledged in the Limitations section. Reporting follows the Strengthening the Reporting of Observational Studies in Epidemiology (STROBE) guideline for cohort studies.

### 2.2 Participants

We reviewed the medical records of all consecutive adult patients (≥18 years) who underwent burr-hole drainage for radiologically confirmed CSDH between January 2018 and December 2023. CSDH was defined on preoperative non-contrast CT as a hypodense, isodense or mixed-density extra-axial collection in the subdural space.

Inclusion criteria: (1) age ≥18 years; (2) preoperative non-contrast CT-confirmed CSDH; and (3) burr-hole drainage as the primary surgical intervention.

Exclusion criteria: (1) acute subdural hematoma, or CSDH developing within 4 weeks of recent intracranial surgery or major head trauma; (2) concurrent intracranial pathology requiring additional surgical intervention (e.g., intracranial tumor, acute intracerebral hemorrhage, traumatic acute subdural hematoma); (3) bilateral CSDH treated with discordant surgical approaches at the same operation (e.g., burr-hole drainage on one side and craniotomy on the contralateral side); and (4) incomplete preoperative radiological data or missing discharge functional outcome.

Patients with bilateral CSDH treated with bilateral burr-hole drainage at the same operation were eligible for inclusion. For these patients, hematoma thickness was recorded as the maximum thickness on either side, and midline shift was recorded as the absolute displacement of the septum pellucidum from the geometric midline irrespective of the direction of shift, reflecting the dominant net mass effect in the presence of asymmetric bilateral disease. Hematoma laterality (right, left or bilateral) and dominant-hemisphere involvement were not analyzed as covariates in the present study, because laterality information was not consistently recorded in a structured electronic field over the study period and could not be retrieved retrospectively with sufficient reliability. We acknowledge this as a source of residual confounding in the Limitations section, and the prognostic relevance of dominant-hemisphere involvement should be addressed in future prospective work.

### 2.3 Radiological measurement

All preoperative CT scans were reviewed independently by two investigators with experience in neurosurgical CT interpretation, blinded to clinical outcome and to each other’s measurements. Disagreements were resolved by consensus discussion in the presence of a senior neurosurgeon. Formal interobserver reliability metrics (e.g., intraclass correlation coefficient) were not calculated in the present analysis; this is acknowledged as a methodological limitation.

Hematoma thickness was measured as the maximal perpendicular distance from the inner table of the skull to the medial margin of the hematoma, on the axial CT slice demonstrating the greatest hematoma extent. We recognize that, on upper cranial/high-convexity axial slices, the inner table of the skull is not perpendicular to the cortical surface and the local cranial curvature may cause direct in-plane axial measurement to overestimate the true cortex-perpendicular thickness. To reduce this source of measurement error, the institutional measurement convention was to select axial slices on which the hematoma margin and the inner table of the skull could be reliably visualized—typically at the level of the basal ganglia, the bodies of the lateral ventricles, or the centrum semiovale—avoiding high-convexity slices whenever possible. When the maximal apparent collection lay on a high-convexity slice, the measurement was taken on the closest mid-axial slice on which the hematoma was still convincingly present. We further acknowledge that single-slice axial thickness is an indirect surrogate for hematoma volume; this issue is explicitly revisited in the Limitations section.

Midline shift was defined as the displacement of the septum pellucidum from the anatomical midline at the level of the foramen of Monro, measured to the nearest 0.1 mm on standard axial CT slices.

Hematoma area was measured on the axial slice demonstrating the greatest hematoma extent using the standard length × width approximation provided by the institutional PACS workstation, where length was the maximum antero-posterior dimension and width the maximum cortex-perpendicular dimension on that slice. We included hematoma area as a descriptive baseline variable to provide complementary two-dimensional information about the spatial extent of the lesion alongside thickness and midline shift. Area was reported in Table 1 only and was not included in the multivariable model, in order to avoid the introduction of strongly collinear radiological covariates (thickness, area, midline shift) and to keep the prognostic model parsimonious; consequently, hematoma area was not interpreted as a free-standing prognostic marker in this study. We further note that direct three-dimensional volumetric measurement (e.g., semi-automated voxel-based segmentation) was not feasible within the constraints of this retrospective single-center analysis, in which CT scans had been acquired with heterogeneous slice thickness over a six-year period and no validated segmentation tool was available institutionally during the study window. The decision to characterize the lesion using thickness and midline shift, rather than volume, therefore reflects both the routine clinical workflow at our center—in which thickness and midline shift are the parameters reported in standard CT reports and used for treatment planning—and the practical constraints of retrospective image data; this is discussed further in the Limitations section.

**Table 1:** Baseline characteristics stratified by discharge functional status.

Variable	Good outcome (mRS 0–2, *n* = 194)	Poor outcome (mRS 3–6, *n* = 66)	*P*
Demographics			
Age, years	71.5 ± 9.2	77.2 ± 7.9	**<0.001**
Male sex	137 (70.6%)	46 (69.7%)	1.000
Comorbidities			
Hypertension	112 (57.7%)	31 (47.0%)	0.169
Diabetes	34 (17.5%)	7 (10.6%)	0.256
Anticoagulant use	66 (34.0%)	28 (42.4%)	0.281
Clinical			
Pre-hospital mRS, median (IQR)	2 (1–3)	2 (2–3)	**0.014**
Pre-hospital mRS ≥ 3	59 (30.4%)	28 (42.4%)	0.102
Radiological burden			
Hematoma thickness, mm	19.3 ± 5.6	20.7 ± 5.5	0.070
Midline shift, mm, median (IQR)	5.9 (3.8–8.4)	6.2 (4.8–8.7)	0.090
Hematoma area, cm^2^	69.9 ± 19.6	74.3 ± 22.3	0.128
Outcome			
Hospital stay, days, median (IQR)	10 (7–13)	11 (7–14)	0.217

Data are presented as mean ± SD, median (IQR), or *n* (%). *P*-values from independent samples *t*-test/Mann–Whitney *U* test for continuous variables and *χ*^2^/Fisher’s exact test for categorical variables. Bold *p*-values indicate *p* < 0.05. Hematoma area is provided as a descriptive baseline variable and was not entered as a covariate in the multivariable model.

### 2.4 Surgical procedure and postoperative management

Burr-hole drainage was performed by attending neurosurgeons of the Department of Neurosurgery according to routine institutional practice. The standard approach consisted of one or two burr holes placed over the area of maximal hematoma thickness, with the number and exact location individualized according to hematoma morphology, the presence of internal septations, the patient’s anatomy, and the operating surgeon’s judgment. Most procedures were performed under local anesthesia with sedation; general anesthesia was used in selected cases at the surgeon’s discretion. After durotomy, the subdural cavity was irrigated with warmed normal saline (0.9% NaCl) until the effluent ran macroscopically clear. A soft Silastic subdural drain was placed in the subdural space at the discretion of the operating surgeon and connected to a closed gravity-dependent drainage system. Drains were typically removed between 24 and 72 h postoperatively, based on drainage volume, character of the effluent and follow-up imaging. Postoperative care followed institutional protocols, including supine bed rest with the head of the bed kept flat or only minimally elevated for the first 24–48 h, gradual mobilization thereafter, and routine early postoperative non-contrast CT to assess residual subdural collection, pneumocephalus and any new intracranial hemorrhage. Antibiotic prophylaxis, perioperative anticoagulant management and the timing of resumption of antithrombotic therapy were individualized according to the treating surgeon and the patient’s underlying medical conditions.

Because of the retrospective nature of the study, complete and uniformly recorded data on all elements of intraoperative technique (e.g., precise number of burr holes, exact volume of irrigation fluid) and postoperative management (e.g., exact mobilization day, drain duration to the hour) were not available for every patient. We did not include these technical details as covariates in the multivariable model; this surgical and postoperative heterogeneity is acknowledged as a limitation and represents a source of residual confounding that should be addressed in prospective protocolized work.

### 2.5 Outcome assessment

The primary outcome was poor discharge functional status, defined *a priori* as an mRS score ≥3 at hospital discharge. The mRS at discharge was assigned by the treating neurosurgical clinical team during routine clinical care, based on the patient’s neurological examination and functional status documented in the medical record. Because outcome assessment was performed during routine clinical care and not by an independent rater, treating clinicians were not formally blinded to preoperative imaging findings or to baseline clinical variables. We cannot exclude assessment bias arising from this lack of blinding; this limitation is acknowledged explicitly in the Limitations section.

The mRS ≥ 3 threshold corresponds to loss of independent ambulation and the need for assistance with activities of daily living, an end point with clear clinical and resource-allocation relevance, and was selected because the mRS was the most consistently and uniformly recorded short-term functional measure in our institutional records during the study period. We acknowledge two important interpretive caveats. First, mRS at discharge captures the patient’s absolute functional status at one cross-sectional time-point and does not directly quantify the change in function from the pre-hematoma baseline to the post-operative state; in particular, a patient with high pre-hospital mRS (for example, mRS 4 due to pre-existing neurological disability) is by definition unable to achieve mRS < 3 at discharge, regardless of any genuine surgical benefit. To partially address this, pre-hospital mRS was prespecified as a covariate in all multivariable models, and the independent associations between radiological variables and the primary outcome therefore reflect estimates conditional on baseline disability rather than on absolute discharge status alone. Second, scales such as the Glasgow Outcome Scale Extended (GOSE) have been preferred in some trauma and CSDH cohorts. GOSE was not consistently recorded in our institutional dataset over the study period and could not be reconstructed reliably from the existing records; we therefore did not perform a GOSE-based analysis. Confirmation of the present findings using longitudinal mRS-based outcomes (e.g., 3- or 6-month follow-up mRS) and/or GOSE remains an important direction for future prospective external validation. Both interpretive limitations are addressed in the Limitations section.

### 2.6 Statistical analysis

Baseline characteristics were compared between outcome groups using independent-samples *t*-tests or Mann–Whitney *U* tests for continuous variables and *χ*^2^ or Fisher’s exact tests for categorical variables, as appropriate. Continuous variables were assessed for normality using the Shapiro–Wilk test.

Univariable logistic regression was performed to estimate crude odds ratios (ORs) with 95% confidence intervals (CIs) for each candidate predictor. A multivariable logistic regression model was then constructed incorporating all prespecified variables: hematoma thickness, midline shift, pre-hospital mRS, age, sex, hypertension, and anticoagulant use. Hematoma area was not included in the multivariable model in order to avoid the introduction of strongly collinear radiological covariates and to maintain a parsimonious specification. Model fit was assessed using the Akaike information criterion (AIC), Nagelkerke pseudo-*R*^2^, and the Hosmer–Lemeshow goodness-of-fit test. Multicollinearity was evaluated using variance inflation factors (VIF), with VIF > 5 considered indicative of problematic collinearity.

To evaluate the functional form of the relationships between continuous radiological variables and the primary outcome, restricted cubic spline (RCS) regression was performed with 4 knots placed at the 5th, 35th, 65th and 95th percentiles of each predictor. The Wald test for nonlinearity was used to assess departure from a linear dose–response relationship. Bootstrap confidence intervals (1,000 resamples) were generated for the spline curves.

The incremental predictive value of variable groups was assessed using nested model comparisons: Model 1 (M1) included radiological burden alone (hematoma thickness and midline shift); Model 2 (M2) added pre-hospital mRS; and Model 3 (M3, full model) further included age, sex, hypertension and anticoagulant use. Likelihood ratio tests were used to compare successive models, and discrimination was quantified by the area under the receiver operating characteristic curve (AUC).

Internal validation was performed using the enhanced bootstrap method (1,000 resamples) to estimate optimism-corrected discrimination and calibration metrics, following the methodology recommended by Steyerberg and Harrell (13). Calibration was evaluated graphically using calibration plots (predicted vs. observed probabilities across deciles) and statistically using the Hosmer–Lemeshow test. Overall predictive accuracy was quantified using the Brier score, where a score of 0 indicates perfect prediction and 0.25 represents non-informative prediction for a 50% event rate. Decision curve analysis (DCA) was performed to evaluate the net clinical benefit of the full model compared with the treat-all and treat-none strategies across a range of clinically relevant threshold probabilities (5–50%) (14). DCA results were interpreted as the net reduction in unnecessary interventions per 100 patients at each threshold.

All analyses were performed using R version 4.3.1 (R Foundation for Statistical Computing, Vienna, Austria) with the rms, pROC, rmda, and ggplot2 packages. A two-sided *p*-value <0.05 was used as the predefined threshold for statistical significance. We did not adjust for multiple comparisons, given the exploratory nature of the analysis.

## 3 Results

### 3.1 Patient characteristics

A total of 260 patients were included in the study (mean age 72.9 ± 9.1 years; 183 [70.4%] male). Sixty-six patients (25.4%) had poor discharge functional status (mRS ≥ 3) at the time of hospital discharge. Baseline characteristics stratified by outcome are presented in Table 1. Patients with poor outcome were significantly older (77.2 ± 7.9 vs. 71.5 ± 9.2 years; *p* < 0.001) and had higher pre-hospital mRS scores (median 2 [IQR 2–3] vs. 2 [1–3]; *p* = 0.014). Both radiological variables were numerically higher in the poor-outcome group, but neither difference met the predefined two-sided *p* < 0.05 threshold: hematoma thickness was 20.7 ± 5.5 mm vs. 19.3 ± 5.6 mm (*p* = 0.070), and midline shift was 6.2 mm (IQR 4.8–8.7) vs. 5.9 mm (3.8–8.4) (*p* = 0.090). There were no statistically significant differences in sex, hypertension, diabetes, anticoagulant use, or length of hospital stay between groups.

### 3.2 Univariable and multivariable logistic regression

**Figure 1:**
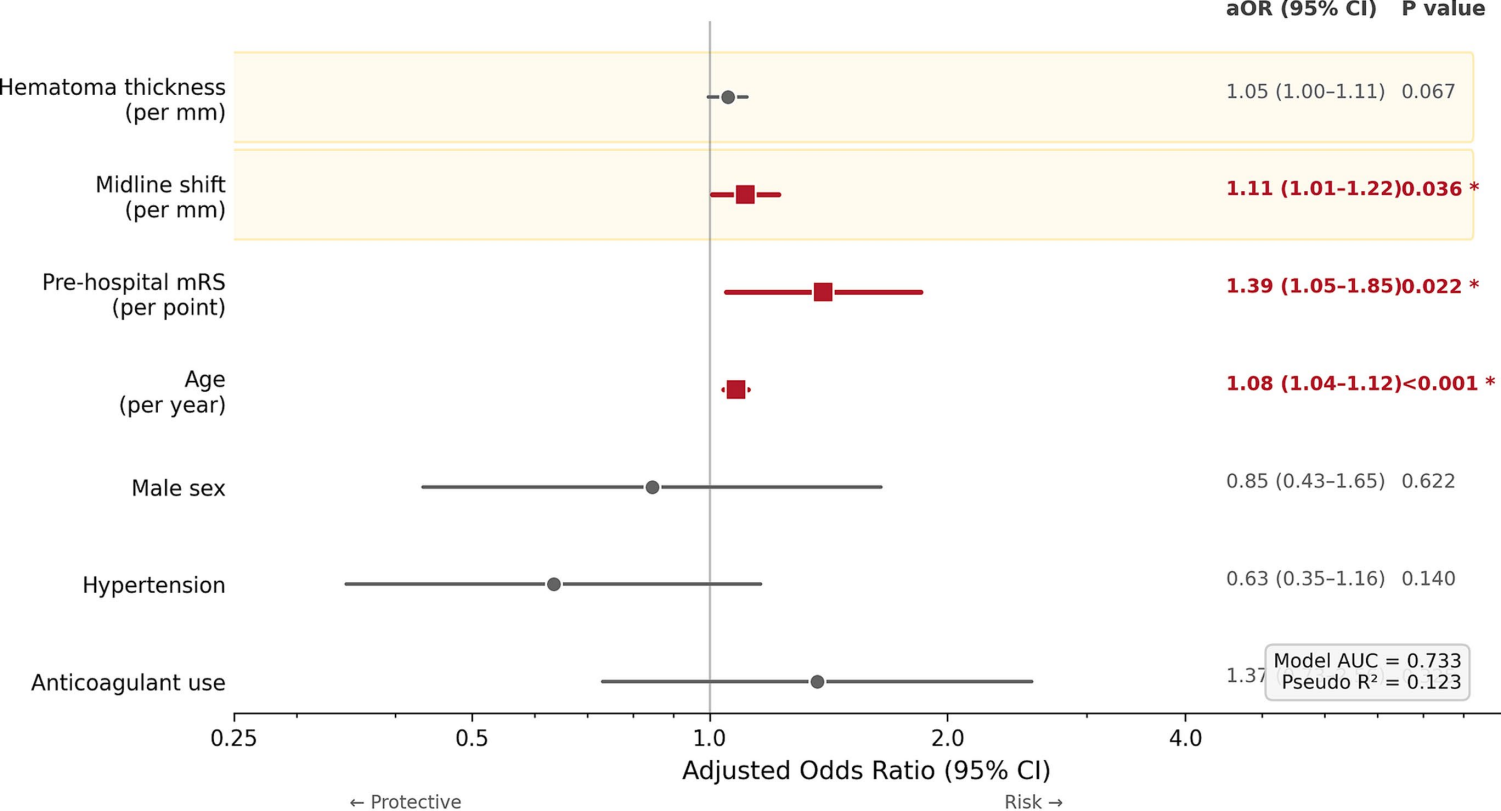
Multivariable logistic regression—forest plot (full model). Adjusted odds ratios with 95% confidence intervals from the full multivariable model (Model 3). Solid square markers indicate variables that met the predefined two-sided *p* < 0.05 threshold. Highlighted rows denote preoperative CT markers (primary exposure variables).

Results of univariable and multivariable logistic regression analyses are shown in Table 2 and Figure 1. In univariable analysis, age (OR 1.07, 95% CI 1.04–1.11; *p* < 0.001) and pre-hospital mRS (OR 1.36, 95% CI 1.05–1.76; *p* = 0.020) were significantly associated with poor discharge functional status. Hematoma thickness (OR 1.05, 95% CI 1.00–1.10; *p* = 0.071) and midline shift (OR 1.09, 95% CI 1.00–1.19; *p* = 0.052) showed numerically positive associations but did not meet the predefined two-sided *p* < 0.05 threshold in univariable analysis.

**Table 2:** Univariable and multivariable logistic regression for poor discharge functional status (mRS ≥ 3).

Variable	Univariable OR (95% CI)	*P*	Multivariable aOR (95% CI)	*P*
Hematoma thickness (per mm)	1.05 (1.00–1.10)	0.071	1.05 (1.00–1.11)	0.067
Midline shift (per mm)	1.09 (1.00–1.19)	0.052	1.11 (1.01–1.22)	**0.036**
Pre-hospital mRS (per point)	1.36 (1.05–1.76)	**0.020**	1.39 (1.05–1.85)	**0.022**
Age (per year)	1.07 (1.04–1.11)	**<0.001**	1.08 (1.04–1.12)	**<0.001**
Male sex	0.96 (0.52–1.76)	0.887	0.85 (0.43–1.65)	0.622
Hypertension	0.65 (0.37–1.14)	0.130	0.63 (0.35–1.16)	0.140
Anticoagulant use	1.43 (0.81–2.53)	0.221	1.37 (0.73–2.56)	0.325

Multivariable model (Model 3) adjusted for all listed variables. OR, odds ratio; aOR, adjusted odds ratio; CI, confidence interval; mRS, modified Rankin Scale. Bold *P*-values indicate *P* < 0.05. Model 3: AIC = 274.4; pseudo-*R*^2^ = 0.123; AUC = 0.733.

In the full multivariable model (Model 3), three variables emerged as independent correlates of poor discharge functional status: age (aOR 1.08, 95% CI 1.04–1.12; *p* < 0.001), pre-hospital mRS (aOR 1.39, 95% CI 1.05–1.85; *p* = 0.022), and midline shift (aOR 1.11, 95% CI 1.01–1.22; *p* = 0.036). Hematoma thickness was numerically positively associated with poor outcome but did not meet the predefined two-sided *p* < 0.05 threshold (aOR 1.05, 95% CI 1.00–1.11; *p* = 0.067). The full model demonstrated acceptable discrimination (AUC = 0.733) and explanatory capacity (pseudo-*R*^2^ = 0.123; AIC = 274.4). Male sex, hypertension and anticoagulant use were not independently associated with the primary outcome in the adjusted analysis. All variance inflation factors were below 1.5, indicating no problematic collinearity among the included variables.

### 3.3 Dose–response analysis

**Figure 2:**
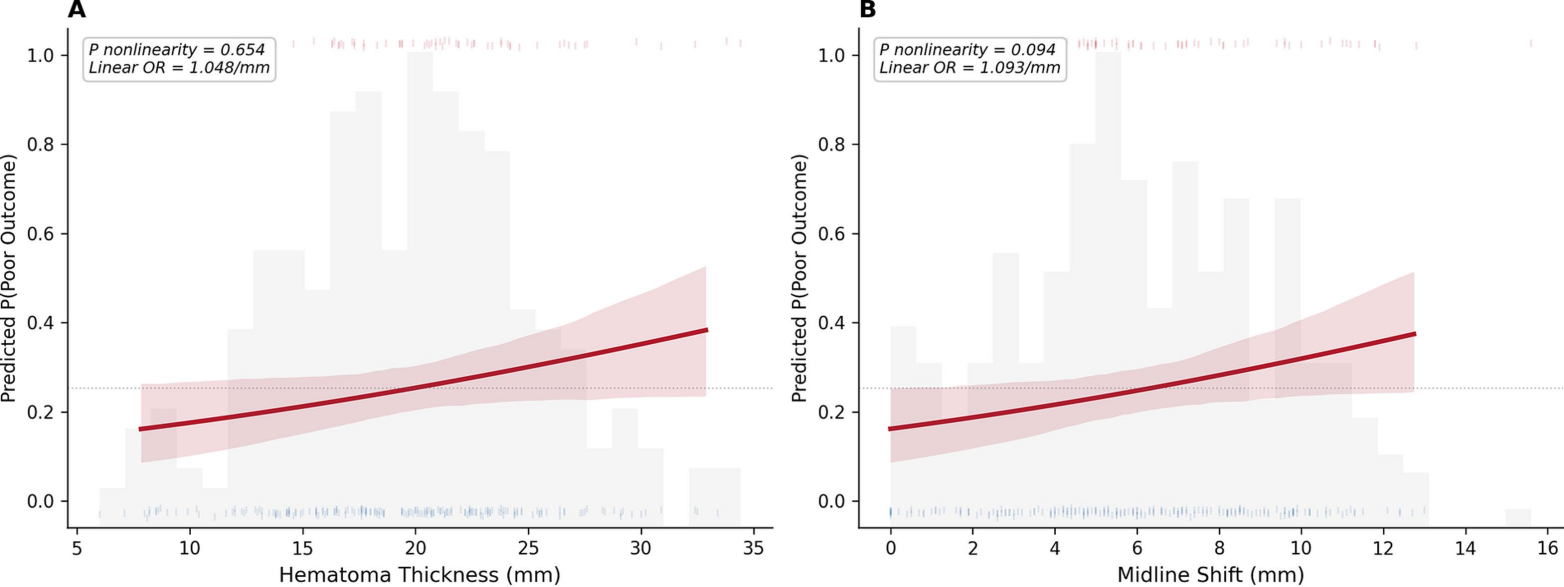
Restricted cubic spline analysis: dose–response relationships. **(A)** Hematoma thickness and **(B)** midline shift versus predicted probability of poor discharge functional status (mRS ≥ 3). Shaded areas represent 95% bootstrap confidence intervals. Rug plots show individual observations. Both relationships are consistent with linearity (*p*non-linearity > 0.05); the test for non-linearity for midline shift returned *p* = 0.094.

Restricted cubic spline analysis was consistent with predominantly linear dose–response relationships for both radiological parameters (Figure 2). For hematoma thickness, the predicted probability of poor outcome increased monotonically from approximately 16% at 7 mm to 38% at 35 mm, with no evidence of nonlinearity (*p* = 0.654; linear OR = 1.048 per mm). For midline shift, the overall relationship was also consistent with a linear pattern (*P*non-linearity = 0.094; linear OR = 1.093 per mm), with predicted probability rising from approximately 16% at 0 mm to 38% at 14 mm. The test for nonlinearity for midline shift returned a value below 0.10 but above the predefined two-sided *p* < 0.05 threshold; we therefore did not reject the null hypothesis of linearity, while acknowledging that a subtle departure from strict linearity at higher midline-shift values cannot be entirely excluded given the sample size. Visual inspection of the spline curve showed no clinically meaningful inflection point, and the confidence band for the nonlinear component broadly overlapped with the linear model. Both curves crossed the overall event rate of 25.4% at approximately 20 mm (hematoma thickness) and 7 mm (midline shift), respectively. The 95% bootstrap confidence intervals widened at extreme values, reflecting reduced sample density in the distribution tails.

### 3.4 Incremental model performance

**Figure 3:**
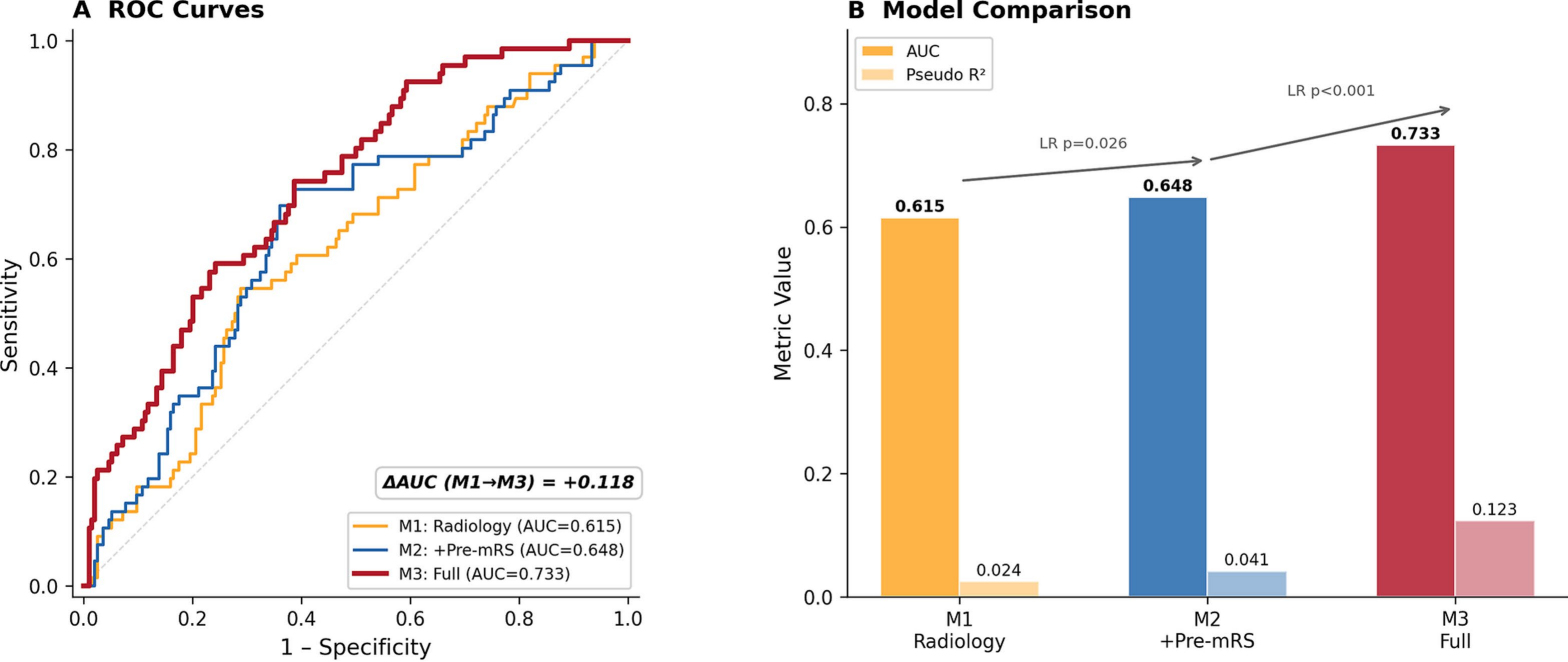
Incremental predictive performance across nested models. **(A)** Receiver operating characteristic curves for three nested models: M1 (preoperative CT markers alone), M2 (M1 + pre-hospital mRS), and M3 (full model adding age, sex, hypertension, and anticoagulant use). **(B)** Model comparison metrics (AUC and pseudo-R_2_) with likelihood ratio test *p*-values for incremental improvement.

The nested model comparison (Figure 3) demonstrated significant incremental improvement in predictive performance. Model 1 (radiological burden alone) achieved an AUC of 0.615 (pseudo-*R*^2^ = 0.024). The addition of pre-hospital mRS (Model 2) improved discrimination (AUC = 0.648; likelihood ratio test *p* = 0.026), and the full model (Model 3) incorporating all clinical covariates yielded the highest discrimination (AUC = 0.733; likelihood ratio test *p* < 0.001 vs. M2). The total AUC improvement from M1 to M3 was 0.118, indicating that while preoperative CT markers provide a foundation for prognostication, the integration of clinical factors substantially enhances predictive accuracy.

### 3.5 Internal validation and calibration

Bootstrap internal validation (1,000 resamples) of the full model (Model 3) yielded an optimism estimate of 0.012 for the AUC, resulting in a bias-corrected AUC of 0.721 (95% CI 0.652–0.790), indicating minimal overfitting. The calibration plot demonstrated good agreement between predicted and observed probabilities across deciles, with a calibration slope of 0.94 and intercept of −0.02, both close to the ideal values of 1 and 0, respectively. The Hosmer–Lemeshow goodness-of-fit test was non-significant (*χ*^2^ = 8.21, df = 8, *p* = 0.42), consistent with adequate calibration. The overall Brier score was 0.168, which compared favorably with the null-model Brier score of 0.190 (scaled Brier score = 0.116), indicating meaningful improvement over a non-informative model.

### 3.6 Decision curve analysis

Decision curve analysis demonstrated that the full model (Model 3) provided positive net clinical benefit compared with the treat-all and treat-none strategies across a wide range of threshold probabilities (approximately 10–45%). At the clinically relevant threshold of 25% (corresponding to the observed event rate), the full model yielded a net benefit of approximately 0.06, equivalent to a net reduction of 6 unnecessary interventions per 100 patients compared with the treat-all approach. Model 1 (radiological burden alone) showed a narrower range of net benefit (approximately 15–30%), further supporting the superiority of the combined model. Above the threshold probability of 45%, all models converged toward the treat-none strategy, consistent with the declining prevalence of predicted high-risk patients.

## 4 Discussion

In this retrospective single-center cohort of 260 patients undergoing burr-hole drainage for CSDH, preoperative midline shift was independently associated with poor discharge functional status after adjustment for established clinical confounders, including age and pre-hospital mRS. Hematoma thickness showed a numerically positive association with the primary outcome but did not meet the predefined two-sided *p* < 0.05 threshold. Restricted cubic spline analysis was consistent with approximately linear dose–response relationships for both hematoma thickness and midline shift, and nested model comparisons indicated that integrating preoperative CT markers with clinical factors improved prognostic discrimination. Internal validation suggested adequate model stability with minimal optimism, and decision curve analysis was consistent with positive net benefit of the combined model across a clinically relevant range of threshold probabilities. These findings should be viewed as exploratory and may help support risk stratification, patient and family counseling, postoperative monitoring, and early rehabilitation planning, rather than treatment selection.

### 4.1 Midline shift as an independent correlate of discharge functional status

Our observation that midline shift was independently associated with poor discharge functional status is broadly compatible with the British multicenter prospective cohort study by Brennan et al. (5), in which radiological burden was associated with poor functional outcome at hospital discharge and by Ro et al. (15), who reported that midline shift was associated with recurrence and outcome in surgically treated CSDH. Conceptually, midline shift may capture the integrated mechanical impact of the subdural collection beyond the simple linear thickness of the hematoma, reflecting the combined effects of mass volume, intracranial compliance, and the degree of cerebral atrophy. Our analysis extends prior reports by characterizing this association as continuous and approximately linear across the observed range, suggesting that each incremental millimeter of midline shift may carry an additional, although modest, increment in risk. This contrasts with the threshold-based pattern at 10 mm proposed by Stanišić et al. (16) in their Norwegian cohort, a discrepancy that may reflect differences in patient populations, surgical timing, outcome definitions and statistical modeling rather than a true biological inconsistency.

### 4.2 Dose–response interpretation

The dose–response analysis warrants nuanced interpretation. For hematoma thickness, the test for nonlinearity was clearly non-significant (*p* = 0.654), and the spline curve was visually consistent with a linear relationship. For midline shift, the test for nonlinearity yielded *p* = 0.094—a value below 0.10 but above the predefined two-sided *p* < 0.05 threshold. We therefore did not reject the null hypothesis of linearity and modeled midline shift as a linear term in the primary analysis; nonetheless, we cannot fully exclude a subtle departure from strict linearity at higher values of midline shift, which a larger sample might have detected. Visual inspection of the spline curve revealed no clinically meaningful inflection point, and the linear model provided an adequate approximation across the observed range. Future studies with substantially larger samples should re-examine whether the relationship is strictly linear or whether a mild threshold effect emerges at higher midline-shift values (17).

### 4.3 Hematoma thickness

Hematoma thickness did not meet the predefined two-sided *p* < 0.05 threshold in the multivariable model (aOR 1.05, 95% CI 1.00–1.11; *p* = 0.067), although the point estimate and the spline curve were consistent with a positive association. A similar pattern, in which radiological size parameters become attenuated after adjustment for age and baseline neurological status, has been documented in earlier multivariable analyses of CSDH outcome (15, 16). One plausible interpretation is that midline shift captures the integrated mechanical impact of the lesion more directly than single-slice axial thickness alone, because midline shift reflects not only hematoma size but also brain compliance, cerebral atrophy and bilateral disease burden (18). We deliberately refrain from inferences about hematoma volume per se, because volume was not directly measured in this study; any extrapolation from thickness or area to volume should be made cautiously, and direct volumetric analysis is an explicit priority for future prospective work.

### 4.4 Clinical predictors and multidimensional assessment

Advanced age and pre-hospital functional status (measured by the mRS) were the strongest independent correlates of poor discharge functional status in our cohort, in agreement with the findings of the British National Health Service study on CSDH outcomes (19). The role of pre-existing disability as a predictor of post-surgical outcome has been increasingly recognized and is biologically plausible: patients with poor baseline function may have diminished neurological reserve and limited capacity for post-operative recovery (20, 21). Recent narrative reviews of CSDH management in older adults have emphasized the importance of assessing baseline functional status in treatment planning (22), and our findings provide quantitative support for incorporating pre-hospital mRS into standardized risk-assessment tools.

The nested model comparison provides a clear quantitative description of the relative contributions of preoperative CT markers and clinical factors. Preoperative CT markers alone demonstrated only modest discriminative ability (AUC = 0.615; pseudo-*R*^2^ = 0.024), consistent with the view that imaging variables capture only one dimension of a fundamentally multifactorial process. The discriminative ability improved substantially with the addition of clinical factors (full-model AUC = 0.733; ΔAUC = +0.118), supporting the use of multidimensional rather than imaging-only assessment in CSDH prognostication. Our findings are broadly compatible with the conclusions of the umbrella review of CSDH outcome predictors by Zhu et al. (23). However, even the full model leaves substantial residual uncertainty at the individual level, as reflected in the moderate AUC and the modest scaled Brier score, and we therefore do not propose that the model in its current form should be used to make individual treatment-allocation decisions.

### 4.5 Internal validation and decision curve analysis

The internal validation analyses strengthen confidence in the model’s apparent stability. The bootstrap-corrected AUC of 0.721 (optimism = 0.012) suggests minimal overfitting in this sample, a recognized concern in logistic regression with moderate event counts. The calibration plot and the non-significant Hosmer–Lemeshow test were consistent with adequate agreement between predicted and observed probabilities. The Brier score of 0.168 represents a modest improvement over the null Brier score of 0.190, and we explicitly note that substantial residual uncertainty at the individual-patient level remains—consistent with the moderate AUC and with the inherently multifactorial nature of CSDH outcomes. Decision curve analysis supported a positive net benefit of the full model, relative to treat-all and treat-none reference strategies, across threshold probabilities of approximately 10–45%; this range corresponds to the band in which the practical question is how intensively to monitor and how proactively to refer for rehabilitation, rather than whether to operate (24).

### 4.6 Mechanistic considerations

Mechanistically, midline shift in CSDH integrates the effects of the hematoma’s mass on a brain whose compliance is modified by age-related atrophy. Progressive accumulation of the subdural collection—driven by neo-membrane angiogenesis and recurrent microhemorrhage—produces mass effect that may compromise regional cerebral perfusion and contribute to secondary neuronal injury (25, 26). Narrative reviews from Japanese and other international groups have proposed incorporating radiological parameters into individualized surgical decision-making for CSDH (27). Our exploratory dose–response data are broadly compatible with this approach but should not be over-interpreted as quantitative evidence supporting any specific operative threshold.

### 4.7 Anticoagulant use

The lack of association between anticoagulant use and discharge functional status in our cohort merits cautious interpretation. Although anticoagulant therapy has been associated with CSDH recurrence in several studies (28), its relationship with functional outcome is less consistent. Fornebo et al. (29) reported in a Norwegian registry that anticoagulant-associated CSDH did not have worse functional outcomes when managed with appropriate perioperative reversal strategies. Our findings are compatible with this view and may suggest that the prognostic impact of anticoagulation is more closely related to recurrence than to immediate discharge functional status; however, this interpretation requires confirmation in studies that capture both recurrence and longitudinal functional outcomes within the same cohort, ideally with stratification by anticoagulant class and by perioperative reversal strategy.

### 4.8 Real-world clinical application

The practical value of this model lies mainly in perioperative planning rather than in selecting a surgical strategy. We propose four concrete uses, all of which fall short of directing the choice of operation. First, in the preoperative consultation, the combination of patient age, pre-hospital mRS and midline shift on the diagnostic CT can be used to give patients and their families a quantitatively grounded, rather than purely qualitative, sense of the expected probability of an mRS ≥ 3 outcome at discharge—supporting counseling and expectation-setting, not the decision to operate. Second, in the immediate postoperative period, patients flagged as higher-risk by the model may be considered for more intensive neurological observation, more proactive postoperative imaging, and earlier multidisciplinary review. Third, the model output can be used at the time of surgical scheduling to identify patients who are likely to benefit from early in-hospital physiotherapy and from early referral to a structured post-discharge rehabilitation pathway, rather than waiting for discharge planning to begin in a reactive fashion. Fourth, at a service-planning level, the case-mix-adjusted predicted-outcome distribution may be used to plan rehabilitation bed and outpatient capacity. None of these uses constitutes a recommendation to apply the model in selecting between surgical strategies, and external validation is required before any of them is adopted as a standard of care.

### 4.9 Limitations

This study has several limitations that should be considered when interpreting the findings. First, the analysis is retrospective, single-center and observational, which limits generalizability and exposes the analysis to unmeasured confounding and selection bias. Second, the study was not prospectively registered and no pre-specified statistical analysis plan was filed before data extraction; although the modeling strategy was specified before formal regression analysis, we cannot fully exclude analytical flexibility, and the results should therefore be regarded as exploratory and hypothesis-generating rather than confirmatory. Third, the primary outcome—mRS at hospital discharge—is a cross-sectional measure of absolute functional status and does not directly capture change from the patient’s pre-hematoma baseline; in particular, patients with high pre-hospital mRS are by definition unable to achieve mRS < 3 at discharge. We attempted to mitigate this by including pre-hospital mRS as a covariate in all multivariable models, but this does not fully resolve the underlying interpretive constraint. Confirmation of the present findings using longitudinal mRS at 3 or 6 months and/or the GOSE is an important direction for prospective work; GOSE was not consistently recorded in our institutional dataset and could not be reconstructed reliably from the existing records. Fourth, the discharge mRS was assigned by the treating neurosurgical team during routine care and was not assessed by a blinded independent rater; we cannot exclude assessment bias from this lack of blinding. Fifth, hematoma laterality (right, left or bilateral, and dominant-hemisphere involvement) was not consistently recorded in a structured field over the study period and was therefore not analyzed as a covariate; this represents a source of residual confounding, since dominant-hemisphere disease is a plausible determinant of the clinical impact of a given thickness or midline shift. Sixth, hematoma volume was not directly measured. The lesion was characterized using single-slice axial thickness and midline shift, which are the parameters reported in routine institutional CT reports, complemented by an exploratory two-dimensional area measurement included for description only; direct three-dimensional volumetric segmentation, with its higher information content, was not feasible in this retrospective dataset and is an explicit priority for future prospective work. Seventh, formal interobserver reliability metrics (e.g., intraclass correlation coefficient) for the radiological measurements were not calculated; disagreements between the two readers were resolved by consensus, but the absence of an ICC limits external comparison. Eighth, complete and uniformly recorded data on intraoperative technique (number of burr holes, irrigation fluid volume, drain placement and duration) and postoperative management (exact mobilization day, postoperative imaging timing) were not available for every patient, and these elements were therefore not included as covariates; the resulting heterogeneity is a source of residual confounding. Ninth, additional clinically relevant variables—including the Glasgow Coma Scale at presentation, hematoma density on CT, the presence of internal septations or membranes, and pneumocephalus on early postoperative imaging—were not consistently available and could not be incorporated into the model (30). Tenth, although bootstrap internal validation suggested limited optimism, external validation in independent multicenter cohorts has not yet been performed and is required before clinical implementation.

### 4.10 Future directions

Future research should focus on developing and externally validating multivariable prediction models that integrate radiological burden, clinical factors and emerging biomarkers (such as inflammatory cytokines or imaging-derived volumetric measurements) in large, multicenter prospective cohorts. Machine-learning approaches may further enhance predictive accuracy by capturing complex nonlinear interactions among predictor variables. Additionally, the development of user-friendly clinical decision-support tools, such as nomograms or web-based calculators, incorporating the key predictors identified in this study would facilitate real-time risk assessment at the bedside, although such tools should not be implemented in routine practice until external validation has been completed.

In addition, the recent publication of phase III randomized trials of middle meningeal artery (MMA) embolization for CSDH [EMBOLISE (31); STEM (32)] has substantially expanded therapeutic options; whether the prognostic factors identified in the present analysis remain operative under MMA-based or MMA-augmented management warrants prospective evaluation in cohorts that include these treatment modalities.

## 5 Conclusion

In this exploratory retrospective cohort study, preoperative midline shift, advanced age, and pre-hospital functional disability were independently associated with poor discharge functional status following burr-hole drainage for chronic subdural hematoma. Dose–response analysis was consistent with predominantly linear relationships between radiological burden and outcome probability, and incremental model comparison demonstrated that combining radiological and clinical factors improved prognostic discrimination relative to radiological variables alone. Internal validation suggested adequate model stability with minimal overfitting, and decision curve analysis suggested potential decision-support value of the combined model across a meaningful range of threshold probabilities. These findings support the integration of midline-shift measurement into multidimensional preoperative risk-stratification and counseling protocols, but should not be used as a stand-alone basis for treatment-selection decisions. External validation in independent multicenter cohorts using longitudinal outcome scales is required before clinical implementation.

## Data Availability

The experimental data used to support the findings of this study are available from the corresponding author upon request.
